# Young Queer Community Members Faced Higher Discrimination in Albania During the COVID-19 Pandemic

**DOI:** 10.7759/cureus.43674

**Published:** 2023-08-17

**Authors:** Elona Gjebrea, Dorina Toçi, Juna Mali, Livia Hoxha

**Affiliations:** 1 Sexual and Reproductive Health and Rights, Albanian Center for Population and Development, Tirana, ALB; 2 Department of Public Health Performance and Chronic Diseases, Institute of Public Health, Tirana, ALB; 3 Epidemiology and Public Health, Albanian Center for Population and Development, Tirana, ALB; 4 Economic Development and Professional Training, Ministry of Finance and Economy, Tirana, ALB

**Keywords:** young people, queer, pandemic, lgbtq+, discrimination, covid-19, albania

## Abstract

Background: The COVID-19 pandemic has hit vulnerable populations harder. In this context, the aim of this study was to assess the negative personal impacts and discrimination experienced by the LGBTQ+ community in Albania during the COVID-19 pandemic.

Methods: This cross-sectional study was carried out in Albania in 2021, as a part of a larger multicenter study conducted by the International Planned Parenthood Federation European Network (IPPF EN). Binary logistic regression was used to assess the likelihood of feeling discriminated, ashamed, or afraid due to sexual orientation, adjusting for main confounding factors.

Results: In total, 279 youngsters aged 14-30 years were included in this study. Of these, 55 participants or 19.7% self-declared as LGBTQ+. Significantly higher proportions of LGBTQ+ were older and of Albanian ethnicity, whereas lower proportions were not married/cohabiting compared to non-LGBTQ+ participants. Significantly higher proportions of LGBTQ+ members have felt discriminated (32.7%), ashamed to discuss about sexual and reproductive health (SRH) issues of concern (32.7%), afraid to express their sexual orientation (45.5%), and lacked privacy to discuss SRH issues with people of trust (36.4%) compared to non-LGBTQ+ participants (5.4%, 15.2%, 4%, and 17.4%, respectively). LGBTQ+ participants were 19.57 times more likely to feel discriminated because of their sexual orientation and 25.05 times more likely to be afraid to express their sexual orientation compared to non-LGBTQ+ participants.

Conclusion: The LGBTQ+ community in Albania was more negatively impacted by the COVID-19 pandemic compared to non-LGBTQ+ participants. The findings should guide future interventions for addressing the needs of the LGBTQ+ community in emergency situations.

## Introduction

LGBTIQ (LGBTQ+) is an acronym that encompasses lesbian, gay, bisexual, transgender, intersex, and queer individuals, representing a diverse range of sexual orientations, gender identities, and intersex variations within the LGBTQ+ community [1], where the + sign represents people with diverse sexual orientation [2]. This inclusive term acknowledges the unique experiences and challenges faced by individuals who do not conform to traditional societal norms regarding sexual orientation or gender identity, promoting acceptance, equality, and human rights for all [3]. LGBTQ+ community members are often exposed to discrimination and hate-motivated violence deeply rooted in homophobic and transphobic attitudes combined with racial discrimination and inadequate legal protection instruments [4]. Preconceived notions and cultural biases about gender and sexual orientation often lead to marginalization and exclusion of LGBTQ+ communities virtually all over the world. Such negative attitudes and behaviors can manifest in different forms of discrimination, such as unequal access to healthcare, housing, employment, and education. For example, a recent systematic review reported that the LGBTQ+ population experiences higher rates of mental health problems, substance abuse, risky sexual behaviors, sexually transmitted infections, and suicide, often due to anti-LGBTQ+ attitudes, bullying, isolation, marginalization, victimization, or social rejection, with poor, sex industry, and ethnic minorities’ LGBTQ+ being more vulnerable [5].

Moreover, LGBTQ+ youth often receive poor healthcare due to stigma, lack of sensitivity and empathy to their needs by the healthcare staff, and low awareness of healthcare providers, among others [6]. Large proportions of LGBTQ+ individuals do not feel comfortable to reveal their sexual identity to their clinicians amidst concerns that such disclosure could negatively affect their interaction with the healthcare provider [6]. Exclusion and other forms of harassment are experienced by the LGBTQ+ community especially in formal education settings where young people gather together for quite a long time, leading to increased mental health problems, lower academic achievement, and lower employment prospects [7]. Thus, the existing international scientific evidence largely suggests that discrimination and negative attitudes against the LGBTQ+ community are accompanied not only by detrimental effects on the overall well-being and productivity of members of this community, but they also might hinder their ability to fully participate in society.

It is very likely for the discrimination against certain population groups to be exacerbated especially during public health emergencies, such as the COVID-19 pandemic [8], which caught the world and virtually all health systems largely unprepared and leaving a heavy toll in terms of health, human psychology, and social, economic, and education systems [9]. Actual vulnerable groups are at risk of becoming even more vulnerable in the event of various public health emergency situations [10]. Hence, the already limited access to healthcare by LGBTQ+ communities due to the predominantly heteronormative attitudes imposed by health professionals under normal situations [11] might further exacerbate during shock events [12]. This implies the need for disaster response efforts to help and protect especially the LGBTQ+ and other vulnerable communities as well, which usually are overrepresented among the at-risk population when an emergency of any type strikes [12]. Hence, in order to appropriately respond to the increased needs of vulnerable communities, first, it is necessary to gather information about the situation of vulnerable population groups and their situation during public health shocks.

This holds true for Albania as well, a small country situated in Southeastern Europe. Similar to the rest of the world, Albania was hit quite hard by the COVID-19 pandemic. As per July 12, 2023, the total number of officially reported COVID-19 deaths in Albania was 3,602 since the start of the pandemic [13], even though the excess mortality during the pandemic years (2020 and 2021) that is largely attributed to COVID-19 is much higher [14,15]. The COVID-19 pandemic was directly responsible for a considerable increase in the poverty rate in Albania, and it is estimated that vulnerable populations were disproportionally hit by the pandemic, including ethnic minorities, families with members with disabilities, women in general, elderly people living alone, and informal workers [16,17]. However, there is no information on the impact of the COVID-19 pandemic on the LGBTQ+ community in Albania, characterized by a still-strong patriarchal mentality and prevailing discriminatory and homophobic attitudes toward the LGBTQ+ individuals [18]. In this context, the aim of this study was to explore how the LGBTQ+ community in Albania was potentially affected by the COVID-19 pandemic in order to inform policy-makers, decision-makers, and field professionals on the situation of this community and potentially guide future efforts to better support this vulnerable group in the eventuality of shock situations.

## Materials and methods

Type of study

The present study is a part of a large cross-sectional multicenter study carried in 2021 by the International Planned Parenthood Federation European Network (IPPH EN) in the framework of the "Youth Voices, Youth Choices" project, carried out in five Balkan countries: Albania, Bosnia and Hercegovina, Bulgaria, Kosovo, and North Macedonia [19]. This large multinational study aimed to better understand the impact of the COVID-19 pandemic on young people with regard to various aspects of sexual and reproductive health services, education, and information and to identify ways to address the SRH needs of young people aged 14-30 years [19]. Separate studies were conducted in each of the countries included in this initiative, and then findings from the participating countries were combined to produce the final report [19].

The actual study is, therefore, based only on the Albanian findings, further expanding on the already published report [20] and viewing the data on the perspective of LGBTQ+ community.

The Albanian Center for Population and Development (ACPD) non-governmental organization, a partner of IPPF, implemented this study in Albania.

Study population and sample size

The study population consisted a sample of young people aged 14-30 years, including men/boys and women/girls alike, recruited for participation on the basis of their potential vulnerability. The study population and sampling techniques have been described in detail elsewhere [20]. In Albania, the study was conducted in four out of 12 regions (prefectures), namely, Tirana, Elbasan, Vlora, and Shkodra, in an attempt to represent all regions of the country (north, center, and south) and to increase the study population’s representativeness [20].

The minimum sample size was calculated via WinPepi software (Windows version of the DOS-based PEPI (Programs for EPIdemiologists), based on the following parameters: alpha error 5%, power of the study 80%, assumed rate in the population 50% (this maximizes the sample size), and acceptable difference 5.5%. These parameters yielded a sample size of 318. We decided to interview more subjects in order to increase the power of the study and to account for any non-responding individuals. As a result, a total of 340 youngsters were included. However, 61 individuals did not prefer to reveal and report their sexual orientation. Therefore, these 61 individuals were excluded from the analysis, thus leaving a total of 279 subjects available for further analysis.

Data collection

The data collection process involved a structured questionnaire that was either self-administered (online) by young people recruited and accepting to be a part of the survey or administered face to face by a skilled interviewer. The online approach was made available in an attempt to attract more youngsters from vulnerable groups who would not like to be identified or to experience a face-to-face interaction with the interviewers. Among the 279 respondents, 245 were interviewed face to face, and 34 completed the questionnaire online. Focus groups discussions were also used to receive more in-depth insights from the participants. A more detailed description of data collection process has been described elsewhere [20].

The questionnaire included several sections asking for the background characteristics of the participants, information, and access to sexual and reproductive health (SRH) services. In particular, the questionnaire contained four questions that explored the negative personal impacts and discrimination experienced by youngsters during the COVID-19 pandemic. These questions are as follows: During the COVID-19 pandemic, when I was looking for any kind of support, information, or service for my SRHR needs, I felt: (1) discriminated against/stigmatized because of who I am; (2) ashamed to discuss an SRH issue that concerned me; (3) more afraid to express my sexual orientation/gender identity; and (4) I did not have the privacy to talk about SRH issues with people I trust or seek information. These four questions (answering options: yes, no, and not applicable) were used in the actual study in order to compare the proportion of LGBTQ+ members who experienced all the above situations against the respective proportions in the self-declared heterosexual study population.

Ethical considerations

The aim and objectives of the study were fully and transparently explained to each of the young people approached. The recruitment and participation of young people across all research phases was carried out in full accordance with all ethical, research, and legal considerations, in line with the ESOMAR code of conduct and local legislation in general and pertaining to the rights of young people [20]. IPPF EN also issued approval for the study.

Statistical analysis

Due to the relatively small number of the self-declared LGBTQ+ participants (*n*=55), we collapsed the categories of some background variables in order to create fewer groups with a decent number of participants that would allow an appropriate statistical analysis. For example, originally, the variable “marital status” had six categories (single, in a relationship, married, divorced but in a relationship, divorced but not in a relationship, and widowed). For our purposes, we created a new two-category variable: married/in a relationship and other marital statuses. The same practice was followed with regard to household head's monthly income and household head's education level.

We reported absolute numbers and respective percentages in order to describe the distribution of the categorical variables included in this survey. Fisher’s exact test was used to compare categorical variables because all tables were in the 2x2 format.

Binary logistic regression analysis was used to calculate the likelihood of experiencing negative personal impact (discrimination, feelings of shame, fear of expressing sexual orientation, and privacy) by sexual orientation status (LGBTQ+ vs. straight/heterosexual individuals). Two models of binary logistic regression were used: in Model 1, we reported crude or unadjusted odds ratios (ORs); in Model 2, we adjusted for the potential confounding effects of age, household head's monthly income, and household head's education level. Accordingly, the respective 95% confidence intervals (CIs) and p-values of the crude (Model 1) and multivariable-adjusted ORs (Model 2) were calculated.

In all cases, a p-value of ≤0.05 was considered as statistically significant. IBM SPSS Statistics for Windows, version 21 (released 2012; IBM Corp., Armonk, New York, United States) was used for all the statistical analyses.

## Results

In total, 279 young persons for whom information on their sexual orientation was available were included in this analysis. Among them, 55 individuals or 19.7% self-declared to belong to the LGBTQ+ community. More than eight in 10 youngsters were 18-30 years old at the time of the survey, 58.5% were female, 13.6% were living in remote areas, 11.8% were of Roma ethnicity, 36.6% were married, 14.7% were in the lowest household monthly income level, and 10.5% declared to live in households where the head of household's education level was primary school or less (Table 1). Significant differences were noticed in the distribution of age, ethnicity, and marital status by sexual orientation, with significantly more LGBTQ+ members being older compared to straight self-declared individuals (96.4% vs. 79%, respectively) or not being married/cohabitating (76.4% vs. 60.3%, respectively) at the time of the survey and significantly fewer LGBTQ+ members being of Roma ethnicity (3.6% vs. 13.8%, respectively). The distribution of other basic demographic and socioeconomic characteristics by sexual orientation was not statistically significant (Table 1).

**Table 1:** General characteristics of the participants by sexual orientation * Absolute number and column percentage (in parentheses); for the total, row percentage. Any discrepancy with the total number is due to missing information. ** P-value according to Fisher’s exact test.

Variable	Total	Sexual orientation		P-value
		Straight	LGBTQ+	
Total	279	224 (80.3%)	55 (19.7%)	
Age				
14-17 years	49 (17.6)*	47 (21.0)	2 (3.6)	0.001 **
18-30 years	230 (82.4)	177 (79.0)	53 (96.4)	
Gender				
Male	115 (41.5)	96 (42.9)	19 (35.8)	0.439
Female	162 (58.5)	128 (57.1)	34 (64.2)	
Living in remote areas				
No	241 (86.4)	196 (87.5)	45 (81.8)	0.277
Yes	38 (13.6)	28 (12.5)	10 (18.2)	
Ethnicity				
Albanian or other	246 (88.2)	193 (86.2)	53 (96.4)	0.036
Roma	33 (11.8)	31 (13.8)	2 (3.6)	
Marital status				
Married/cohabiting	102 (36.6)	89 (39.7)	13 (23.6)	0.029
Other	177 (63.4)	135 (60.3)	42 (76.4)	
Household monthly income				
Lowest income level	31 (14.7)	23 (13.1)	8 (22.9)	0.187
Higher income level	180 (85.3)	153 (86.9)	27 (77.1)	
Household head education level				
Primary school or less	28 (10.5)	26 (12.2)	2 (3.8)	0.083
Secondary school or higher	238 (89.5)	187 (87.8)	51 (96.2)	

Table 2 shows the distribution of participants by how they feel when seeking any kind of support, information, or service regarding their SRHR needs during the COVID-19 pandemic. In general, 10.8% of the responding participants have felt discriminated against or stigmatized because of who they are during the pandemic, 18.6% were ashamed to discuss about an SRH issue concerning them during the pandemic, 12.2% felt more afraid to express their sexual orientation/gender identity, and 21.1% felt not having the privacy to talk about SRH issues with people they trust. In all cases, compared to their straight/heterosexual peers, significantly higher proportions of the LGBTQ+ community felt discriminated against (5.4% vs. 32.7%, respectively), were ashamed to discuss about an SRH issue of interest (15.2% vs. 32.7%, respectively), were more afraid to express their sexual identity (4% vs. 45.5%, respectively), and felt to not have the privacy to talk about SRH issues with people of trust (17.4% vs. 36.4%, respectively) (Table 2).

**Table 2:** Distribution of the participants based on their feeling when seeking any kind of support, information, or service regarding their sexual and reproductive health (SRH) needs during the COVID-19 pandemic * Absolute number and column percentage (in parentheses). Any discrepancy with the total number is due to missing information. ** P-value according to the chi-square test.

Variable	Total	Sexual orientation		P-value
		Straight	LGBTQ+	
Discriminated against or stigmatized because of who I am				
Yes	30 (10.8)*	12 (5.4)	18 (32.7)	<0.001 **
No	152 (54.5)	133 (59.4)	19 (34.5)	
Did not apply	97 (34.8)	79 (35.3)	18 (32.7)	
Ashamed to discuss about a SRH issue which concerned me				
Yes	52 (18.6)	34 (15.2)	18 (32.7)	0.011
No	160 (57.3)	133 (59.4)	27 (49.1)	
Did not apply	67 (24.0)	57 (25.4)	10 (18.2)	
More afraid to express my sexual orientation/gender identity				
Yes	34 (12.2)	9 (4.0)	25 (45.5)	<0.001
No	153 (54.8)	137 61.2()	16 (29.1)	
Did not apply	92 (33.0)	78 (34.8)	14 (25.5)	
I did not have the privacy to talk about SRH issues with people I trust or seek information				
Yes	59 (21.1)	39 (17.4)	20 (36.4)	0.006
No	130 (46.6)	112 (50.0)	18 (32.7)	
Did not apply	90 (32.3)	73 (32.6)	17 (30.9)	

LGBTQ+ members were 19.57 times more likely to feel discriminate or stigmatized because of who they are during the COVID-19 pandemic compared to their straight self-declared peers, and this difference is highly significant (p<0.001) (Table 3, Model 2). Similarly, LGBTQ+ members were 25.05 times more likely to express their sexual orientation during the COVID-19 pandemic compared to their straight self-declared peers (p<0.001). In the univariate analysis, LGBTQ+ members were 2.61 times more likely to feel ashamed to discuss about an SRH issue of concern for them and 3.91 times more likely to feel to not have privacy when talking about such issues with persons of trust compared to their heterosexual peers, and these differences were significant (p<0.05). However, after adjusting for the potential confounding effect of age, household income, and household head's education level, these differences turned not significant, but even these associations kept their direction, and the last one had borderline significance (p=0.068) (Table 3, Model 2).

**Table 3:** Association of feeling discriminated/stigmatized against because of who one is (results of binary logistic regression) * Crude unadjusted odds ratios (OR) from binary logistic regression. ** ORs adjusted for age, household income, and household head's education level. *** 95% confidence interval (CI) for OR. ref.: reference category

Variable	Model 1 – Univariate analysis*		Model 2 – Multivariable adjusted analysis**	
	OR (95% CI) ***	P value	OR (95% CI)	P value
Odds ratios of feeling discriminated or stigmatized because of who I am				
Sexual orientation				
Straight	1.00 (ref.)	<0.001	1.00 (ref.)	<0.001
LGBTQ+	10.50 (4.38-25.17)		19.57 (5.57-68.72)	
Odds ratios of feeling ashamed to discuss about an SRH issue that concerned me				
Sexual orientation				
Straight	1.00 (ref.)	0.008	1.00 (ref.)	0.145
LGBTQ+	2.61 (1.29-5.28)		1.96 (0.79-4.84)	
Odds ratios of feeling more afraid to express my sexual orientation/ gender identity				
Sexual orientation				
Straight	1.00 (ref.)	<0.001	1.00 (ref.)	<0.001
LGBTQ+	23.76 (9.45-59.76)		25.05 (7.47-83.98)	
Odds ratios of feeling to not have the privacy to talk about SRH issues with people I trust or seek information				
Sexual orientation				
Straight	1.00 (ref.)	0.002	1.00 (ref.)	0.068
LGBTQ+	3.91 (1.53-6.65)		3.38 (0.94-6.02)	

## Discussion

This is the first study reporting in extensive details on the situation of the LGBTQ+ community members with regard to the negative personal impacts and discrimination experienced by them during the COVID-19 pandemic in Albania. The findings revealed that significantly higher proportions of the LGBTQ+ community experienced feelings of shame, fear of expressing sexual orientation, and privacy concerns during the COVID-19 pandemic compared to their straight/heterosexual self-declared peers. More specifically, LGBTQ+ members were about 20 times significantly more likely of feeling discriminated because of who they are and about 25 times significantly more likely to be afraid of expressing their sexual orientation/gender identity during the pandemic compared to heterosexual individuals. LGBTQ+ members also were more likely to feel ashamed of discussing about SRH issues of concern and to feel a lack of privacy to talk about SRH issues with people they trust, compared to heterosexual individuals. 

These findings unveil the difficult situation that the LGBTQ+ community finds itself in Albania surrounded by a society largely dominated by negative attitudes toward LGBTQ+ individuals. A recently published study reported that 94% of Albanian citizens would not support their child if he/she belonged to the LGBTQ+ community, 53% believe that LGBTQ+ individuals should not be free to live their life as they wish, and 30% are against same-sex relationships, placing Albania as the most homophobic country in the region [18]. Such attitudes are widespread among teachers and employers as well [18]. It is very likely that such attitudes were further accentuated and exacerbated during the COVID-19 pandemic as the intensity of emotion expressed in focus group discussions, and sensed by us, was very probably an indication that vulnerable groups have become more aware of discriminatory practices as a result of the pandemic [20]. This is supported by the fact that, especially in Albania and North Macedonia, members of the LGBTQ+ community have a widespread and deep mistrust in health institutions probably rooted on the discrimination they have experienced in the past [19]. Because COVID-19 contributed to the disruption of many health services, such a mistrust built on past experiences might have further contributed to deteriorating the situation of the LGBTQ+ community during the pandemic in a disproportionate way compared to their heterosexual peers.

The negative effects of COVID-19 measures and restrictions on the LGBTQ+ community have been observed in many other countries as well. A recent study that assessed how COVID-19 has impacted the health, social, and occupational areas of functioning of the LGBTQ+ community reported that the worsening of physical health, mental health, financial stability, ability to meet basic needs and social connectedness had affected significantly higher proportions of the LGBTQ+ study group compared to heterosexual individuals, implying that such negative effects have affected the LGBTQ+ community at a higher rate compared to the heterosexual population [21]. Another study reported that young LGBTQ+ individuals in the 7th, 9th, and 11th grades in Thailand exhibited a worse mental health status (higher prevalence of depressive symptoms and suicidal ideation), higher prevalence of alcohol use, higher prevalence of experiences of violence, and significantly higher engagement in sexual risky behaviors and illegal drug use [22]. Another study that included a sample of college students aged 18+ in the USA reported that LGBTQ+ individuals that had experienced identity-related victimization since the start of the COVID-19 pandemic were 4.2 times more likely to experience moderate to severe psychological distress compared to LGBTQ+ individuals, reporting no identity-related victimization; in addition, the level of psychological stress during the pandemic is considerably higher than that before the pandemic, implying the need for appropriate tailored interventions designed by health, medical, and education practitioners to mitigate the negative mental health effects of the pandemic among LGBTQ+ young adults [23].

Furthermore, the authors identified social isolation (the most widespread direct consequence of COVID-19 restrictive measures) to be a significant predictor of psychological stress among the LGBTQ+ community, highlighting the need for social support of LGTBQ+ young adults [23]. The negative effects of the COVID-19 pandemic are not felt only among young LGBTQ+ members. For example, a systematic review that aimed to review the impact of COVID-19 on LGBTQIA+ older adults' (65 years and older) health, including risk and protective factors, reported that mental health deteriorated for about 40% of them, whereas the frequency of social interaction had reduced for more than 90% of LGBTQ+ individuals, with negative health effects being deteriorated by perceived homophobia and heterosexism within health institutions [24], implying that negative consequences affect all LGBTQ+ individuals regardless of their age. Other research has highlighted that the LGBTQ+ youth was hit harder than the non-LGBTQ+ youth by the COVID-19 pandemic [25].

The discrimination and stigma toward the LGBTQ+ community, in place even before the pandemic [26], were very likely exacerbated during the COVID-19 pandemic, both from government representatives and healthcare providers [27]. The global cross-sectional analysis of experiences of violence and discrimination among LGBTQ+ individuals during the COVID-19 pandemic, by Adamson and colleagues [27], reported that a staggering 84.4% of the LGBTQ+ community members declared to have experienced the same or more discrimination and violence than before the COVID-19 pandemic due to their sexual orientation, gender identity, gender expression, or sex characteristics. The authors concluded that around the world, government officials, policymakers, and healthcare providers continue to exert systemic discrimination and fail to prevent violence against the LGBTQ+ community [27]. Furthermore, the relief efforts aimed to protect the population from new vulnerabilities associated with COVID-19 did not impact the LGBTQ+ community which, on the contrary, found itself in an even more disadvantaged position than before the pandemic [27]. The prospects of being discriminated against based on one’s sexual orientation might push the LGBTQ+ members to not use healthcare or other governmental services where they think the potential for discrimination is higher [28,29] or has more engagement in risky and self-harming behaviors [29], further exacerbating their already vulnerable and disadvantageous situation.

It seems that the COVID-19 pandemic has hit hard especially the LGBTQ+ communities across the world. We also demonstrated that a similar situation occurred in Albania during the pandemic as well, with significantly higher proportions of the LGBTQ+ community experiencing feelings of shame, fear of expressing sexual orientation, and privacy issues, compared to heterosexual youngsters. Even though there are no previous studies to report on the prevalence of experiencing discrimination among the LGBTQ+ community before the pandemic, we believe that the excess proportion of the LGBTQ+ individuals admitting to face discrimination during the pandemic (compared to the prevalence among heterosexual young participants) could be largely attributed to the COVID-19 dynamics. In this light, there is need to conduct future research in order to confirm and/or replicate the findings of the actual study. 

This study has several limitations. Its cross-sectional nature does not allow to draw conclusions about the temporality of events; thus, the causality of relationships is not implied. This study employed a non-random selection of participants, thus limiting the potential for the generalization of findings. Furthermore, the relatively small study population further limits the generalization potential. Lastly, the information bias cannot be ruled out as LGBTQ+ participants might have been especially reluctant to answer frankly. Nonetheless, this study has some strong points as well. This is the first study reporting on the negative personal impacts and discrimination experienced by the LGBTQ+ young community in Albania during the COVID-19 pandemic. In addition, we employed robust statistical methods to determine and highlight the likelihood of the LGBTQ+ individuals to experience discrimination, controlling for confounding effects of several independent factors. Lastly, this study could serve as a baseline effort against which to compare the results of other future studies that shall explore similar research aspects.

## Conclusions

Significantly higher proportions of LGBTQ+ were older and of Albanian ethnicity, whereas lower proportions were not married/cohabiting compared to non-LGBTQ+ participants. Our findings demonstrated that the LGBTQ+ community in Albania faced higher discrimination in Albania during the COVID-19 pandemic compared to their non-LGBTQ+ peers. This might be reflected in a higher mistrust to access health institutions and, consequently, in a further deterioration of the situation of this community. It is of paramount important to collect information on the LGBTQ+ community discrimination levels and the factors perpetrating and maintaining, as well as their needs, in order to reduce existing disparities. Our findings could serve policy-makers, decision-makers, and interested professionals for better addressing the concerns and needs of the Albanian LGBTQ+ community in emergency situations.
